# Supplementary material for: Polygenic scores capture genetic modification of the adiposity-cardiometabolic risk factor relationship
Source: Cell Genom. 2025 Nov 25;6(3):101075. doi: 10.1016/j.xgen.2025.101075 (PMC12985365; doi:10.1016/j.xgen.2025.101075)
Supplement: Document S1. Figures S1–S10 and Tables S1–S9 [file mmc1.pdf]

Cell Genomics, Volume *6*

Supplemental information

Polygenic scores capture genetic modification of the adiposity-cardiometabolic risk factor relationship

Kenneth E. Westerman, Julie E. Gervis, Luke J. O'Connor, Miriam S. Udler, and Alisa K. Manning

Supplemental Figures

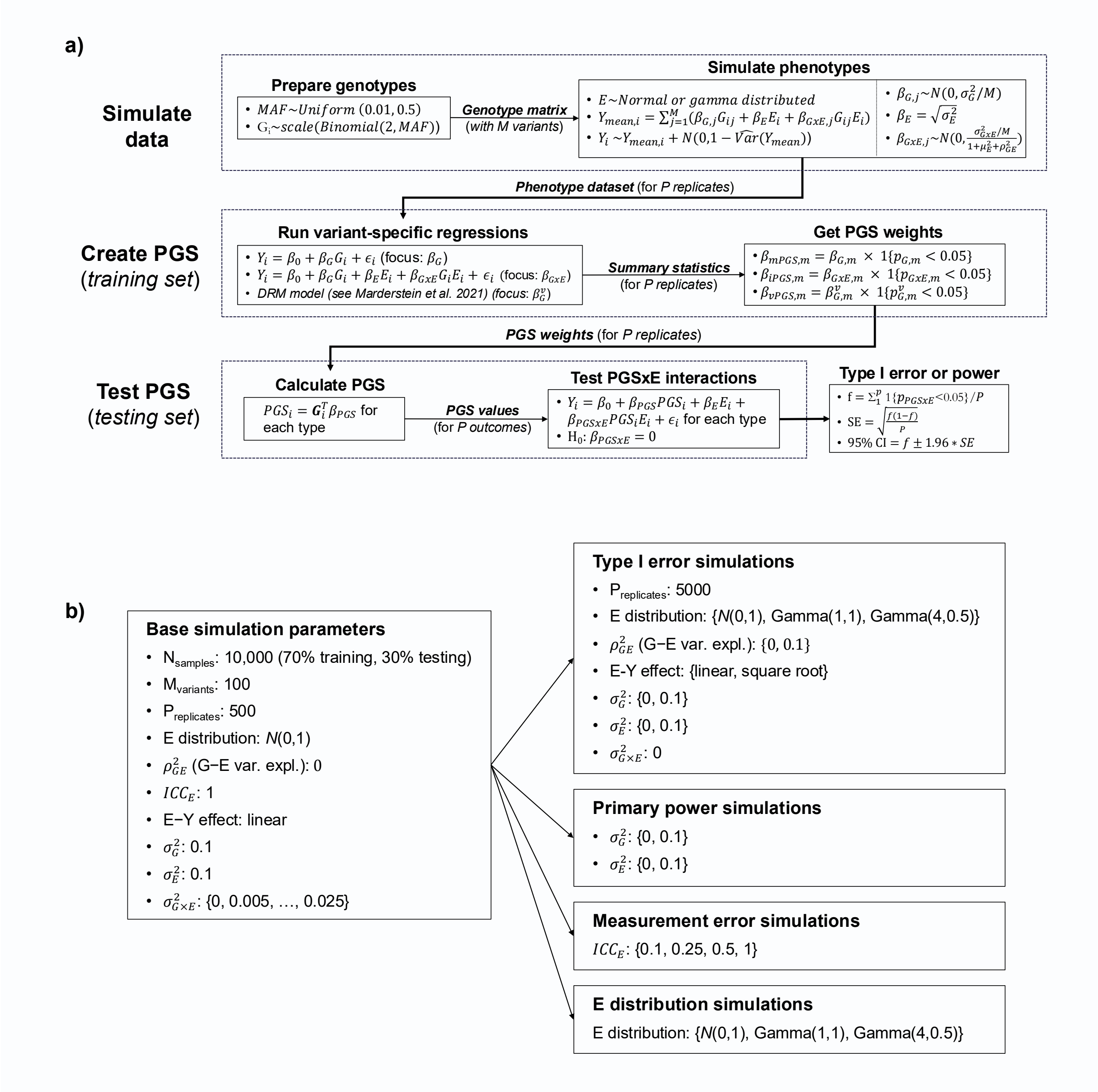

Supplementary Figure S1, related to Figure 2: Simulation study overview. (a) Simulation pipeline (see Methods for additional details). (b) Simulation parameters defining scenarios for type I error and power calculation. Base simulation parameters (left box) apply unless overridden by scenario-specific parameters (right boxes). Gamma distributions are parameterized as (shape, scale). G: NxM genotype matrix; E: NxP exposure matrix; Y: NxP outcome matrix.

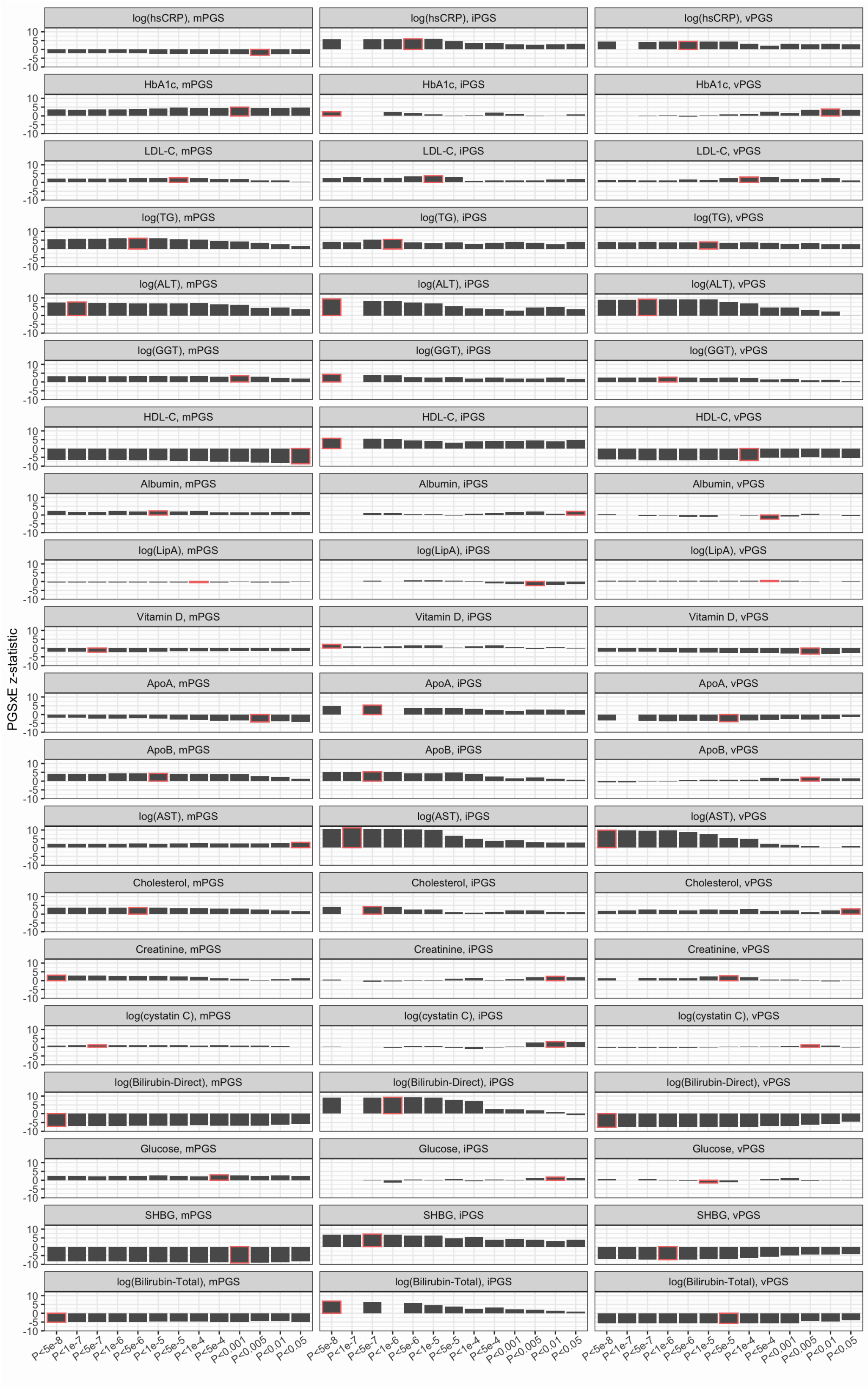

PGSxE z-statistic
log(hsCRP), mPGS
log(hsCRP), iPGS
log(hsCRP), vPGS
HbA1c, mPGS
HbA1c, iPGS
HbA1c, vPGS
LDL-C, mPGS
LDL-C, iPGS
LDL-C, vPGS
log(TG), mPGS
log(TG), iPGS
log(TG), vPGS
log(ALT), mPGS
log(ALT), iPGS
log(ALT), vPGS
log(GGT), mPGS
log(GGT), iPGS
log(GGT), vPGS
HDL-C, mPGS
HDL-C, iPGS
HDL-C, vPGS
Albumin, mPGS
Albumin, iPGS
Albumin, vPGS
log(LipA), mPGS
log(LipA), iPGS
log(LipA), vPGS
Vitamin D, mPGS
Vitamin D, iPGS
Vitamin D, vPGS
ApoA, mPGS
ApoA, iPGS
ApoA, vPGS
ApoB, mPGS
ApoB, iPGS
ApoB, vPGS
log(AST), mPGS
log(AST), iPGS
log(AST), vPGS
Cholesterol, mPGS
Cholesterol, iPGS
Cholesterol, vPGS
Creatinine, mPGS
Creatinine, iPGS
Creatinine, vPGS
log(cystatin C), mPGS
log(cystatin C), iPGS
log(cystatin C), vPGS
log(Bilirubin-Direct), mPGS
log(Bilirubin-Direct), iPGS
log(Bilirubin-Direct), vPGS
Glucose, mPGS
Glucose, iPGS
Glucose, vPGS
SHBG, mPGS
SHBG, iPGS
SHBG, vPGS
log(Bilirubin-Total), mPGS
log(Bilirubin-Total), iPGS
log(Bilirubin-Total), vPGS
10
5
0
-5
-10
P<5e-8
P<1e-7
P<5e-7
P<1e-6
P<5e-6
P<1e-5
P<5e-5
P<1e-4
P<5e-4
P<0.001
P<0.005
P<0.01
P<0.05

Supplementary Figure S2, related to Figure 3: Optimization of PGS for PGSxE interaction across all CRFs. Red boxes indicate the optimal *p*-value threshold selected (to maximize the magnitude of the xPGS×BMI z-statistic).

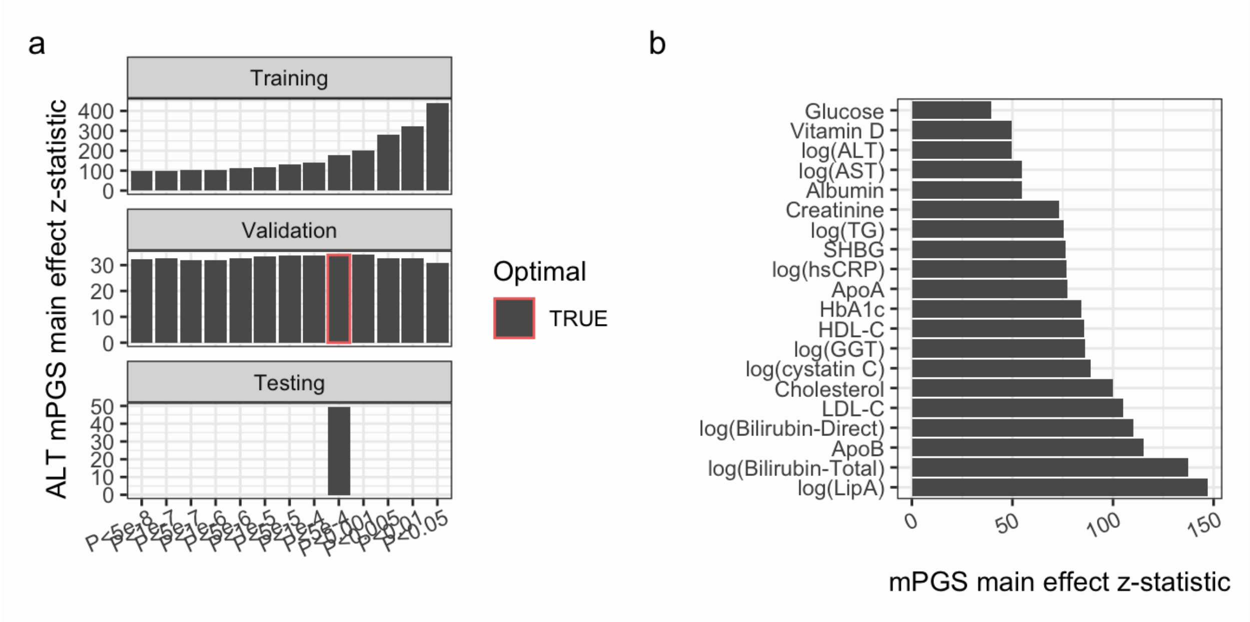

Supplementary Figure S3, related to Figure 3: Marginal effects of standard PGS as a positive control for the PGS development pipeline. (a) mPGS main effects in the training, optimization, and testing subsets. Red outline indicates the best-performing *p*-value threshold in the optimization subset. (b) Marginal mPGS effect z-statistics for each biomarker in the testing subset.

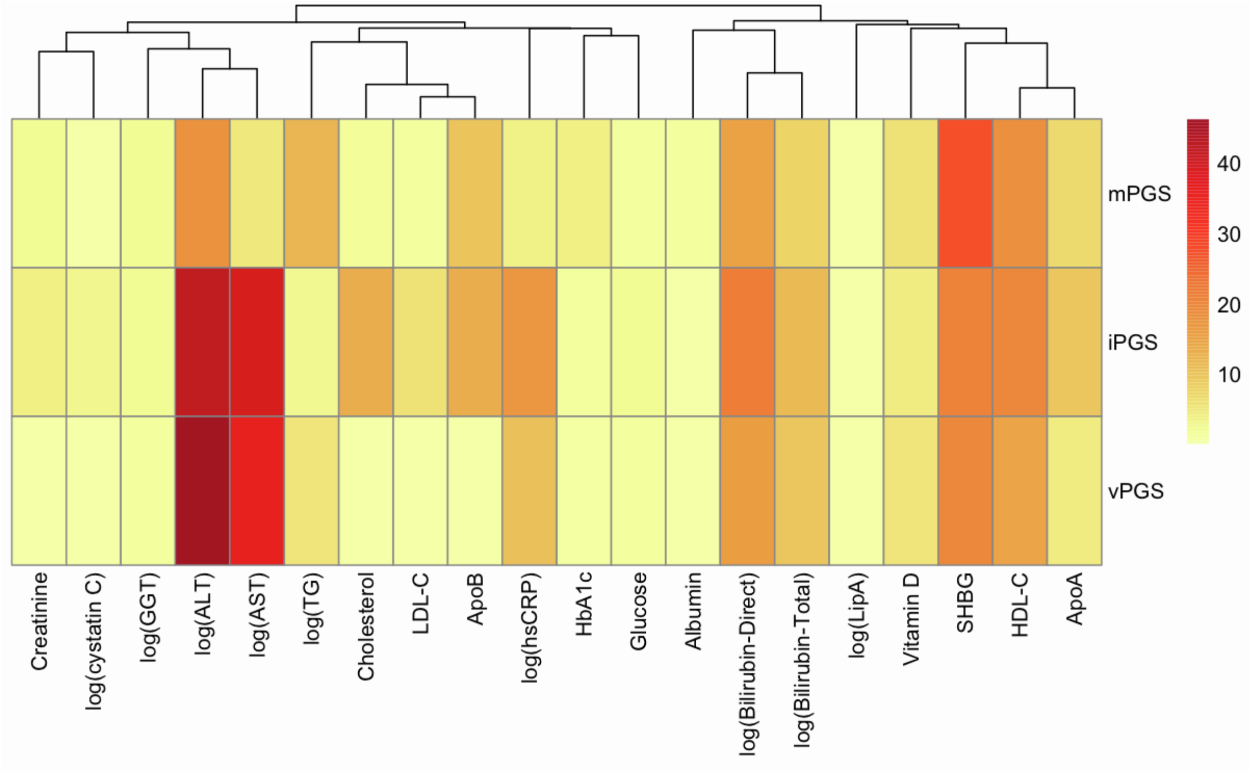

Supplementary Figure S4, related to Figure 3: Heatmap of PGS performance (as measured by -$\log_{10}(p_{PGS \times BMI})$) by CRF. CRFs are ordered based on a hierarchical clustering (Euclidean distance and complete linkage) of their phenotypic values in the UKB training set after mean imputation of missing values.

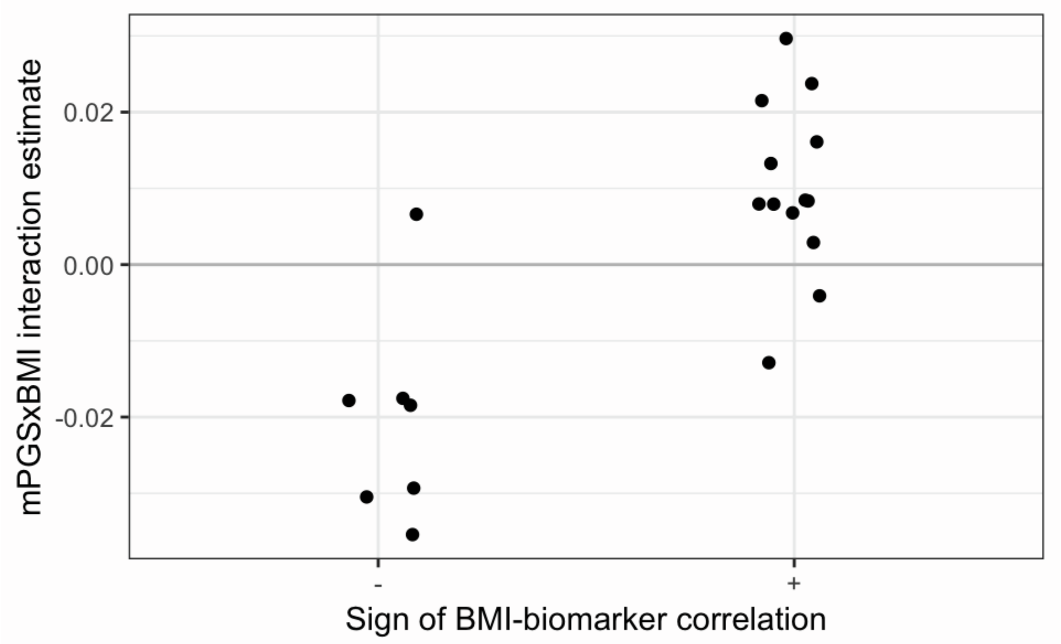

Supplementary Figure S5, related to Figure 3: Relationship between negative mPGSxBMI estimates and directionality of the BMI-CRF relationship (with implications for the amplification model of GxE

interactions). Interaction effects between the mPGS and BMI (*y*-axis) are plotted against the raw sign of the BMI-CRF correlation (*x*-axis).

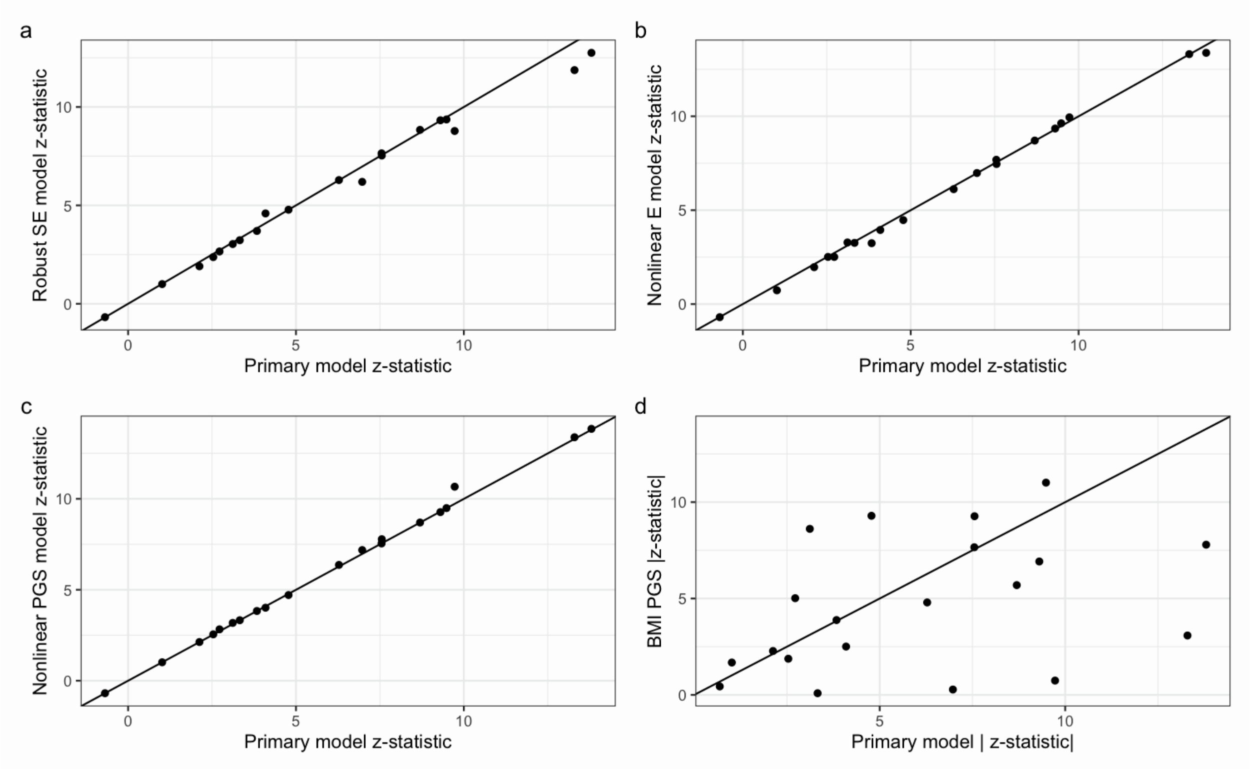

Supplementary Figure S6, related to Figure 3: Sensitivity analyses addressing possible artifactual PGSxBMI in the UKB testing set. Plotted against primary iPGS model z-statistics for each of the 20 CRFs are z-statistics from models (a) using robust standard errors, (b) including a squared term for the BMI main effect, (c) including a squared term for the PGS main effect, and (d) replacing the iPGS with an mPGS for BMI (i.e., developed using BMI as the outcome, rather than the exposure), plotting absolute values of the z-statistics for . Solid lines denote $x = y$.

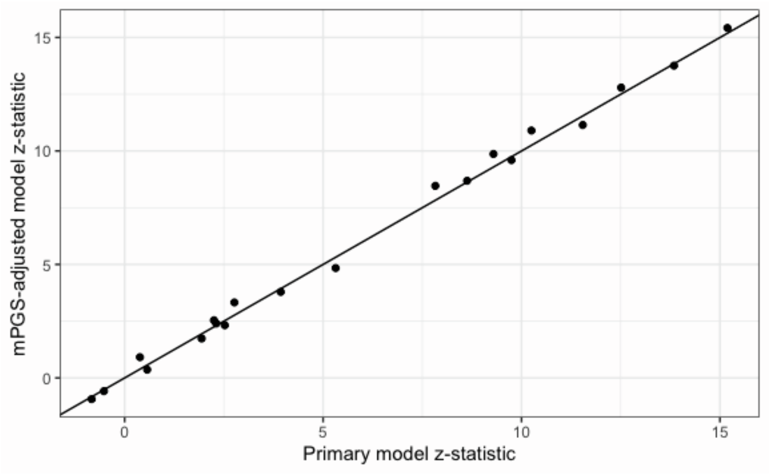

Supplementary Figure S7, related to Figure 3: Sensitivity analysis addressing the relevance of adjusting for mPGS to reduce the standard errors of estimates. For each of the 20 CRFs, z-statistics from

iPGS interaction models are plotted, either from the primary model (*x*-axis) or additionally adjusting for an mPGS for that biomarker (*y*-axis). Solid line denotes *x* = *y*.

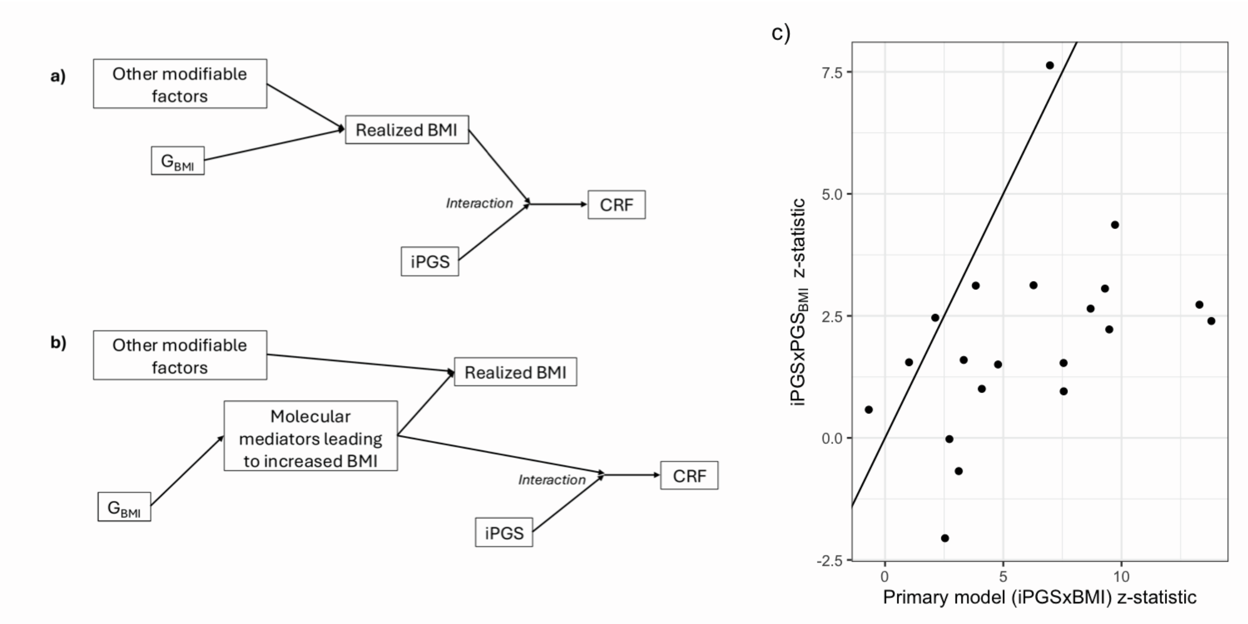

Supplementary Figure S8, related to Figure 3: Sensitivity analysis addressing the genetic underpinnings of BMI. a,b) Conceptual model describing the scenario in which replacing measured ("realized") BMI with a PGS reflecting its genetic influences (here, G_{BMI}) would or would not increase power for discovery of interactions with the iPGS. In (a), the iPGS interaction involves involves BMI itself, such that the upstream "cause" of BMI does not change the interaction strength. In (b), the iPGS interaction involves molecular mediators of the G-BMI relationship, such that replacing BMI with a genetic anchor might reveal a stronger interaction. c) Comparison of z-statistics across all CRFs from the primary iPGS tests (*x*-axis) or identical tests replacing BMI with an mPGS for BMI (*y*-axis). Solid line denotes *x* = *y*.

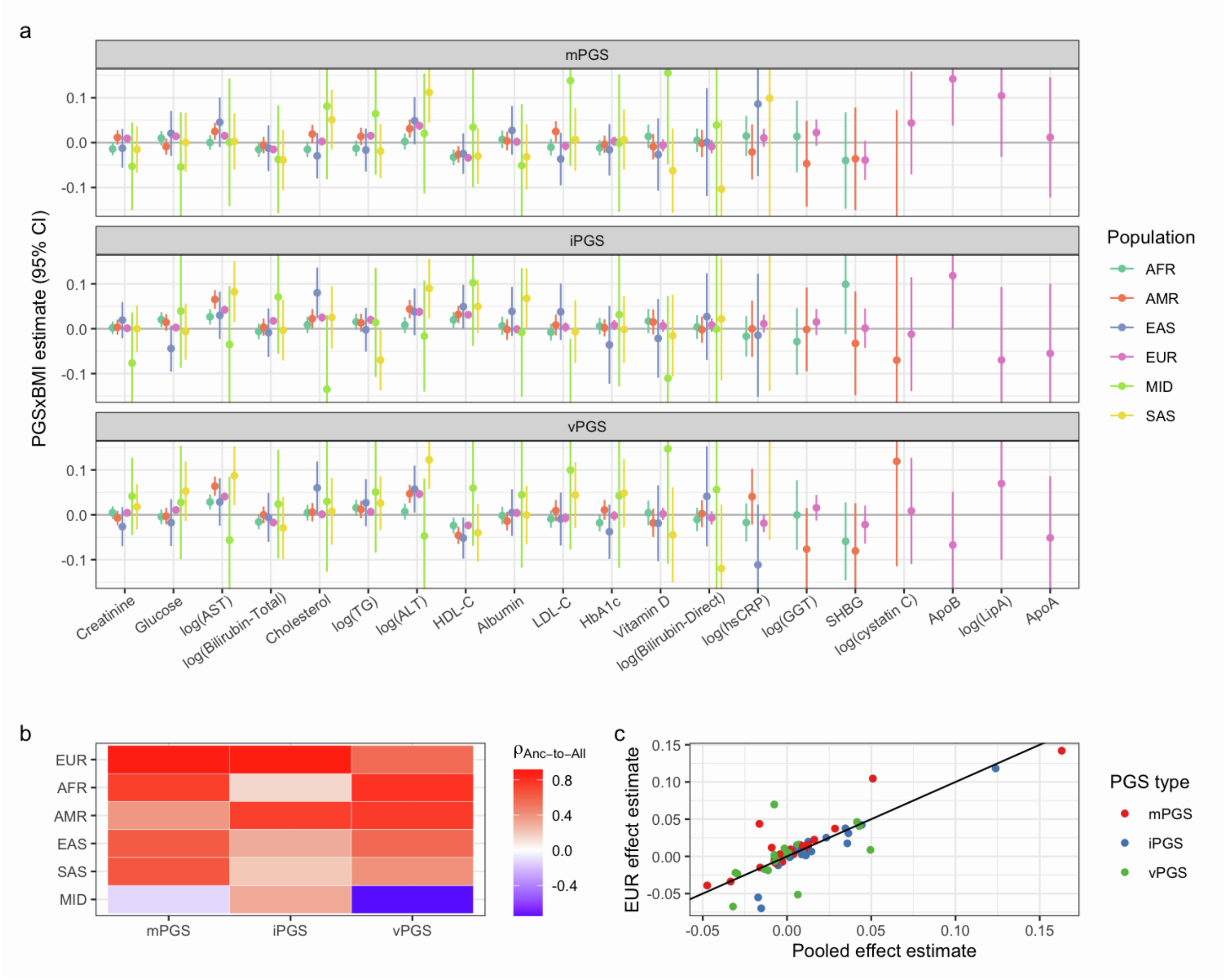

Supplementary Figure S9, related to Figure 4: Ancestry-specific PGS×BMI results in All of Us. (a) Full set of ancestry-specific results (points are shown for ancestry-CRF combinations with sample size >100). (b) Heatmap shows Pearson correlations between PGS×BMI effect estimates in the pooled dataset ("all") and specific genetically inferred ancestry groups (calculated across the 20 CRFs, with the same sample size filter as in (a)). (c) European ancestry-specific interaction effects are plotted against those from regressions using the full, pooled dataset (with gPC-based ancestry pre-adjustment). Colors correspond to PGS type.

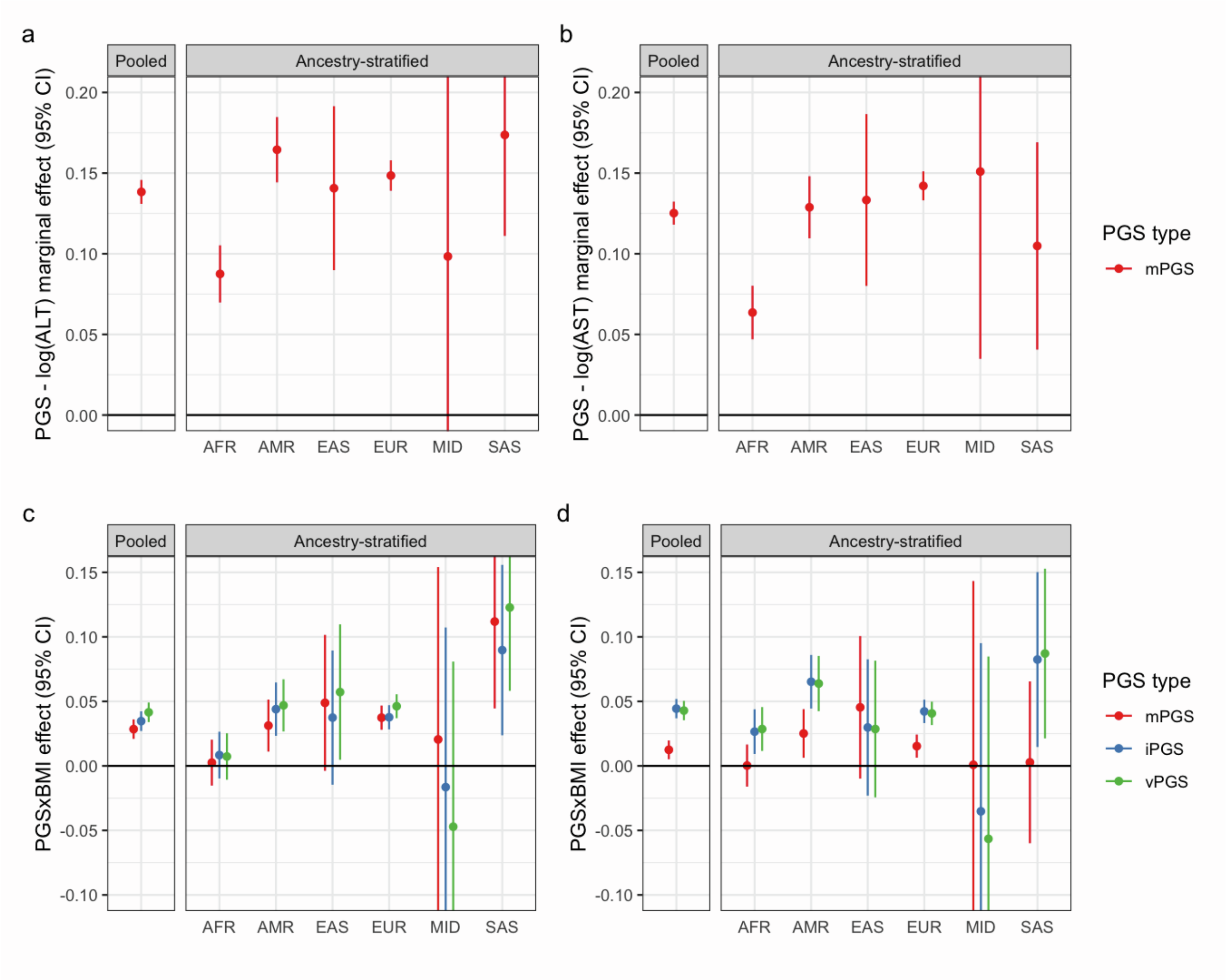

Supplementary Figure S10, related to Figure 5: Ancestry-specific replication of marginal and interaction effects in All of Us. (a-b) Standardized marginal mPGS effects on log(ALT) (a) and log(AST) (b) in the full pooled dataset and genetic ancestry groups. (c-d) Standardized iPGS×BMI interaction effects on log(ALT) (c) and log(AST) (d) in the full pooled dataset and genetic ancestry groups.

Supplemental Tables

Supp. Table S1: UKB population description, related to the STAR Methods

	Training set	Validation set	Testing set
N	253536	36220	72439
Sex, % female	55.9	55.7	55.9
Age, years	56.0 (8.1)	56.0 (8.1)	56.0 (8.1)
BMI, kg/m^2	27.1 (4.5)	27.1 (4.5)	27.1 (4.5)

Supp. Table S2: UKB biomarker metadata, related to the STAR Methods

Cardiometabolic risk factor	Units	UKB Field	Log-transformed
hsCRP	mg/L	30710	Yes
HbA1c	mmol/mol	30750	No
LDL-C	mmol/L	30780	No
TG	mmol/L	30870	Yes
ALT	U/L	30620	Yes
GGT	U/L	30730	Yes
HDL-C	mmol/L	30760	No
Albumin	g/L	30600	No
LipA	nmol/L	30790	Yes
Vitamin D	nmol/L	30890	No
ApoA	g/L	30630	No
ApoB	g/L	30640	No
AST	U/L	30650	Yes
Cholesterol	mmol/L	30690	No
Creatinine	umol/L	30700	No
cystatin C	mg/L	30720	Yes
Bilirubin-Direct	umol/L	30660	Yes
Glucose	mmol/L	30740	No
SHBG	nmol/L	30830	No
Bilirubin-Total	umol/L	30840	Yes

Supp. Table S3: UKB PGSxBMI results, related to the STAR Methods

Biomarker	PGS Type	Optimal threshold	PGSxBMI estimate	Z-statistic	P-interaction
alt_log	vpgs	Pt_5e-07	0.049	14.404	5.75e-47
alt_log	ipgs	Pt_5e-08	0.0472	13.8	2.92e-43
ast_log	ipgs	Pt_1e-07	0.0485	13.296	2.74e-40
ast_log	vpgs	Pt_5e-08	0.0465	12.72	5.03e-37
shbg	mpgs	Pt_0.001	-0.0354	-11.049	2.35e-28
bilirubin_dir_log	ipgs	Pt_1e-06	0.0347	9.727	2.42e-22
shbg	ipgs	Pt_5e-07	0.0317	9.482	2.57e-21
hdl	ipgs	Pt_5e-08	0.0309	9.302	1.43e-20
shbg	vpgs	Pt_1e-06	-0.0307	-9.23	2.79e-20
hdl	mpgs	Pt_0.05	-0.0305	-8.966	3.15e-19
alt_log	mpgs	Pt_1e-07	0.0297	8.75	2.18e-18
hscrp_log	ipgs	Pt_5e-06	0.0295	8.694	3.57e-18
bilirubin_dir_log	vpgs	Pt_5e-08	-0.0297	-8.263	1.45e-16
bilirubin_dir_log	mpgs	Pt_5e-08	-0.0293	-8.089	6.15e-16
hdl	vpgs	Pt_0.0001	-0.0269	-7.949	1.91e-15
apoB_statinadj	ipgs	Pt_5e-07	0.0273	7.556	4.2e-14
chol_statinadj	ipgs	Pt_5e-07	0.0275	7.55	4.41e-14
tg_log	mpgs	Pt_5e-06	0.0237	7.015	2.33e-12
bilirubin_tot_log	ipgs	Pt_5e-08	0.0236	6.973	3.14e-12
hscrp_log	vpgs	Pt_5e-06	0.0225	6.688	2.28e-11
apoB_statinadj	mpgs	Pt_1e-05	0.0215	6.397	1.59e-10
apoA	ipgs	Pt_5e-07	0.0226	6.28	3.41e-10
bilirubin_tot_log	vpgs	Pt_5e-05	-0.0201	-6.259	3.9e-10
bilirubin_tot_log	mpgs	Pt_5e-08	-0.0178	-5.559	2.72e-08
apoA	mpgs	Pt_0.005	-0.0184	-5.248	1.54e-07
ldl_statinadj	ipgs	Pt_1e-05	0.0175	4.778	1.77e-06
vitD	mpgs	Pt_5e-07	-0.0175	-4.754	2e-06
tg_log	vpgs	Pt_1e-05	0.0161	4.656	3.23e-06
vitD	vpgs	Pt_0.005	-0.0182	-4.568	4.94e-06
ast_log	mpgs	Pt_0.05	0.0161	4.475	7.65e-06
vitD	ipgs	Pt_5e-08	0.0153	4.093	4.26e-05
apoA	vpgs	Pt_5e-05	-0.0144	-4.072	4.67e-05
hba1c	mpgs	Pt_0.001	0.0133	3.869	0.000109
creatinine	ipgs	Pt_0.01	0.0124	3.836	0.000125
hscrp_log	mpgs	Pt_0.005	-0.0129	-3.743	0.000182
cysC_log	ipgs	Pt_0.01	0.0109	3.326	0.00088

tg_log	ipgs	Pt_1e-06	0.011	3.116	0.00183
creatinine	mpgs	Pt_5e-08	0.0084	2.828	0.00469
ggt_log	ipgs	Pt_5e-08	0.0094	2.722	0.00649
ggt_log	mpgs	Pt_0.001	0.0085	2.556	0.0106
glu	ipgs	Pt_0.01	0.0101	2.537	0.0112
chol_statinadj	mpgs	Pt_5e-06	0.008	2.307	0.0211
hba1c	ipgs	Pt_5e-08	0.0076	2.124	0.0337
ggt_log	vpgs	Pt_1e-06	0.0069	2.04	0.0414
glu	mpgs	Pt_0.0005	0.0079	2.002	0.0453
ldl_statinadj	mpgs	Pt_5e-05	0.0068	1.983	0.0473
glu	vpgs	Pt_1e-05	0.0084	1.922	0.0547
alb	mpgs	Pt_1e-05	0.0066	1.736	0.0826
hba1c	vpgs	Pt_0.01	0.007	1.4	0.162
lipA_log	mpgs	Pt_0.0001	-0.0041	-1.069	0.285
alb	ipgs	Pt_0.05	0.004	1.014	0.311
creatinine	vpgs	Pt_5e-05	0.0031	0.995	0.32
cysC_log	mpgs	Pt_5e-07	0.0029	0.912	0.362
apoB_statinadj	vpgs	Pt_0.005	-0.0029	-0.77	0.442
lipA_log	ipgs	Pt_0.005	-0.0029	-0.688	0.491
cysC_log	vpgs	Pt_0.005	-0.0022	-0.664	0.506
alb	vpgs	Pt_0.0005	-0.0025	-0.66	0.509
lipA_log	vpgs	Pt_0.0005	-0.0025	-0.609	0.543
chol_statinadj	vpgs	Pt_0.05	0.0022	0.596	0.551
ldl_statinadj	vpgs	Pt_0.0001	-9.00E-04	-0.234	0.815

Supp. Table S4: UKB LDSC results, related to the STAR Methods

CRF	rho_g	P-value
alt_log	0.51	5.7e-14
ggt_log	0.5	8.5e-8
hba1c	0.44	1.5e-6
tg_log	0.37	4.5e-6
ast_log	0.33	3.1e-5
creatinine	0.41	2e-4
shbg	-0.25	2e-4
hdl	-0.39	5e-4
vitD	-0.44	0.029
chol_statinadj	0.26	0.077
alb	0.19	0.08
apoB_statinadj	0.13	0.23
ldl_statinadj	0.11	0.27
bilirubin_tot_log	-0.06	0.6
hscrp_log	-0.09	0.63
apoA	-0.06	0.73
bilirubin_dir_log	-0.05	0.76
lipA_log	NA	NA
cysC_log	NA	NA
glu	NA	NA

Supp. Table S5: AoU population, related to the STAR Methods

N	90386
Sex, % female	Female (59258; 65.6%), Male (29343; 32.5%), No answer (819; 0.9%), Other (966; 1.1%)
Age, years	54.3 (16.3)
Genetically predicted ancestry	AFR (17361; 19.2%), AMR (13330; 14.7%), EAS (1912; 2.1%), EUR (56237; 62.2%), MID (379; 0.4%), SAS (1167; 1.3%)
BMI, kg/m^2	29.7 (7.2)

Supp. Table S6: AoU biomarker metadata, related to the STAR Methods

Biomarker	Concept names	Valid units
alb	Albumin [Mass/volume] in Serum or Plasma; Albumin [Mass/volume] in Serum or Plasma by Electrophoresis	gram per liter
alt	Alanine aminotransferase [Enzymatic activity/volume] in Serum or Plasma	unit per liter; No matching concept; U/L; IU/L; u/L
apoA	Apolipoprotein A-I [Mass/volume] in Serum or Plasma	milligram per deciliter; mg/dL
apoB	Apolipoprotein B [Mass/volume] in Serum or Plasma	milligram per deciliter; mg/dL
ast	Aspartate aminotransferase [Enzymatic activity/volume] in Serum or Plasma	unit per liter; No matching concept; U/L; IU/L; u/L
bilirubin_dir	Bilirubin.direct [Mass/volume] in Serum or Plasma	milligram per deciliter; mg/dL
bilirubin_tot	Bilirubin.total [Mass/volume] in Serum or Plasma	milligram per deciliter; mg/dL
chol	Cholesterol [Mass/volume] in Serum or Plasma	milligram per deciliter; mg/dL
creatinine	Creatinine [Mass/volume] in Serum or Plasma	milligram per deciliter; mg/dL
cysC	Cystatin C [Mass/volume] in Serum or Plasma	milligram per liter; mg/L
ggt	Gamma glutamyl transferase [Enzymatic activity/volume] in Serum or Plasma	unit per liter; No matching concept; U/L; IU/L; u/L
glu	Glucose [Mass/volume] in Serum or Plasma	milligram per deciliter; mg/dL
hba1c	Hemoglobin A1c/Hemoglobin.total in Blood; Hemoglobin A1c/Hemoglobin.total in Blood by HPLC	% of total; Percent; Percentage unit; percent; percent hemoglobin; percent hemoglobin A1c
hdl	Cholesterol in HDL [Mass/volume] in Serum or Plasma; Cholesterol in HDL [Mass/volume] in Serum or Plasma by Electrophoresis	milligram per deciliter; mg/dL
hscrp	C reactive protein [Mass/volume] in Serum or Plasma; C reactive protein [Mass/volume] in Serum or Plasma by High sensitivity method	milligram per deciliter; mg/dL
ldl	Cholesterol in LDL [Mass/volume] in Serum or Plasma by calculation; Cholesterol in LDL [Mass/volume] in Serum or Plasma by Direct assay; Cholesterol in LDL [Mass/volume] in Serum or Plasma by Electrophoresis	milligram per deciliter; mg/dL
lipA	Lipoprotein a [Mass/volume] in Serum or Plasma	milligram per deciliter; mg/dL
shbg	Sex hormone binding globulin [Moles/volume] in Serum or Plasma	nanomole per liter; nmol/L
tg	Triglyceride [Mass/volume] in Serum or Plasma	milligram per deciliter; mg/dL
vitD	25-hydroxyvitamin D3 [Mass/volume] in Serum or Plasma; Cholecalciferol (Vit D3) [Mass/volume] in Serum or Plasma	nanogram per milliliter

Supp. Table S7: AoU PGSxBMI results, related to the STAR Methods

Biomarker	PGS Type	# variants (UKB score)	# variants (AoU calculation)	PGSxBMI estimate	Z-statistic	P-interaction	N
hscrp_log	mpgs	15462	4820	0.0125	1.434	0.152	11278
hscrp_log	ipgs	22	21	0.0075	0.84	0.401	11278
hscrp_log	vpgs	57	54	-0.0113	-1.287	0.198	11278
hba1c	mpgs	7145	4790	-0.0041	-0.994	0.32	47119
hba1c	ipgs	1	1	0.0059	1.332	0.183	47119
hba1c	vpgs	21396	4884	-0.0074	-1.929	0.0537	47119
ldl	mpgs	2281	2198	-0.0028	-0.656	0.512	53241
ldl	ipgs	29	27	0.0039	0.899	0.369	53241
ldl	vpgs	413	405	-0.0066	-1.578	0.115	53241
tg_log	mpgs	1242	1193	0.0077	2.07	0.0385	62937
tg_log	ipgs	8	8	0.0126	3.298	0.000973	62937
tg_log	vpgs	115	112	0.0024	0.641	0.522	62937
alt_log	mpgs	331	318	0.0285	7.418	1.2e-13	62634
alt_log	ipgs	11	11	0.0347	8.911	5.17e-19	62634
alt_log	vpgs	40	40	0.0415	10.752	6.1e-27	62634
ggt_log	mpgs	6753	4822	0.0162	1.243	0.214	5523
ggt_log	ipgs	3	3	0.0059	0.46	0.645	5523
ggt_log	vpgs	148	145	0.0066	0.521	0.602	5523
hdl	mpgs	69295	4815	-0.0335	-9.66	4.64e-22	62350
hdl	ipgs	5	5	0.0364	10.451	1.52e-25	62350
hdl	vpgs	536	530	-0.0294	-8.494	2.04e-17	62350
alb	mpgs	875	841	0.002	0.499	0.617	60512
alb	ipgs	53784	4841	0.0016	0.408	0.684	60512
alb	vpgs	1291	1253	6e-04	0.156	0.876	60512
lipA_log	mpgs	568	564	0.0509	0.942	0.347	383
lipA_log	ipgs	7103	4854	-0.0153	-0.217	0.828	383
lipA_log	vpgs	1850	1812	-0.0076	-0.109	0.914	383
vitD	mpgs	301	289	-0.0075	-1.406	0.16	27549
vitD	ipgs	2	2	0.0145	2.777	0.00549	27549
vitD	vpgs	12332	4848	-0.0075	-1.447	0.148	27549
apoA	mpgs	15064	4816	-0.0091	-0.16	0.873	346
apoA	ipgs	8	8	-0.0171	-0.254	0.8	346
apoA	vpgs	208	200	0.0065	0.106	0.915	346
apoB	mpgs	1678	1615	0.163	3.278	0.00114	437
apoB	ipgs	10	10	0.1239	2.148	0.0323	437
apoB	vpgs	11830	4858	-0.0321	-0.512	0.609	437

ast_log	mpgs	64536	4815	0.0125	3.366	0.000764	69777
ast_log	ipgs	11	11	0.0444	11.656	2.28e-31	69777
ast_log	vpgs	15	15	0.0429	11.231	3.03e-29	69777
chol	mpgs	1647	1588	0.0038	1.001	0.317	63304
chol	ipgs	12	12	0.0233	5.959	2.56e-09	63304
chol	vpgs	76234	4882	-1.00E-04	-0.022	0.983	63304
creatinine	mpgs	618	588	0.0021	0.702	0.483	76321
creatinine	ipgs	13974	4844	0.0015	0.494	0.621	76321
creatinine	vpgs	173	170	0.001	0.332	0.74	76321
cysC_log	mpgs	992	958	-0.0164	-0.356	0.722	573
cysC_log	ipgs	13624	4857	-0.0052	-0.116	0.908	573
cysC_log	vpgs	11259	4868	0.0495	1.103	0.271	573
bilirubin_dir_log	mpgs	269	254	-0.0069	-1.112	0.266	26342
bilirubin_dir_log	ipgs	13	13	0.0084	1.4	0.162	26342
bilirubin_dir_log	vpgs	168	161	-0.0072	-1.161	0.246	26342
bilirubin_tot_log	mpgs	379	355	-0.0159	-4.488	7.19e-06	67624
bilirubin_tot_log	ipgs	5	5	0.0358	10.349	4.4e-25	67624
bilirubin_tot_log	vpgs	357	345	-0.0132	-3.755	0.000174	67624
glu	mpgs	1896	1833	0.0093	2.67	0.00758	75604
glu	ipgs	13536	4883	0.0087	2.514	0.0119	75604
glu	vpgs	46	45	-0.0016	-0.473	0.636	75604
shbg	mpgs	5461	4810	-0.0474	-2.495	0.0127	2211
shbg	ipgs	15	15	0.0112	0.587	0.557	2211
shbg	vpgs	85	82	-0.0306	-1.74	0.0821	2211

Supp. Table S8: AoU vs. UKB results, related to the STAR Methods

Biomarker	PGS Type	PT_threshold	UKB estimate	UKB P	AoU estimate	AoU P	AoU N
alt_log	vpgs	Pt_5e-07	0.049	5.75e-47	0.0415	6.1e-27	62634
alt_log	ipgs	Pt_5e-08	0.0472	2.92e-43	0.0347	5.17e-19	62634
ast_log	ipgs	Pt_1e-07	0.0485	2.74e-40	0.0444	2.28e-31	69777
ast_log	vpgs	Pt_5e-08	0.0465	5.03e-37	0.0429	3.03e-29	69777
shbg	mpgs	Pt_0.001	-0.0354	2.35e-28	-0.0474	0.0127	2211
bilirubin_dir_log	ipgs	Pt_1e-06	0.0347	2.42e-22	0.0084	0.162	26342
shbg	ipgs	Pt_5e-07	0.0317	2.57e-21	0.0112	0.557	2211
hdl	ipgs	Pt_5e-08	0.0309	1.43e-20	0.0364	1.52e-25	62350
shbg	vpgs	Pt_1e-06	-0.0307	2.79e-20	-0.0306	0.0821	2211
hdl	mpgs	Pt_0.05	-0.0305	3.15e-19	-0.0335	4.64e-22	62350
alt_log	mpgs	Pt_1e-07	0.0297	2.18e-18	0.0285	1.2e-13	62634
hscrp_log	ipgs	Pt_5e-06	0.0295	3.57e-18	0.0075	0.401	11278
bilirubin_dir_log	vpgs	Pt_5e-08	-0.0297	1.45e-16	-0.0072	0.246	26342
bilirubin_dir_log	mpgs	Pt_5e-08	-0.0293	6.15e-16	-0.0069	0.266	26342
hdl	vpgs	Pt_0.0001	-0.0269	1.91e-15	-0.0294	2.04e-17	62350
apoB	ipgs	Pt_5e-07	0.0273	4.2e-14	0.1239	0.0323	437
chol	ipgs	Pt_5e-07	0.0275	4.41e-14	0.0233	2.56e-09	63304
tg_log	mpgs	Pt_5e-06	0.0237	2.33e-12	0.0077	0.0385	62937
bilirubin_tot_log	ipgs	Pt_5e-08	0.0236	3.14e-12	0.0358	4.4e-25	67624
hscrp_log	vpgs	Pt_5e-06	0.0225	2.28e-11	-0.0113	0.198	11278
apoB	mpgs	Pt_1e-05	0.0215	1.59e-10	0.163	0.00114	437
apoA	ipgs	Pt_5e-07	0.0226	3.41e-10	-0.0171	0.8	346
bilirubin_tot_log	vpgs	Pt_5e-05	-0.0201	3.9e-10	-0.0132	0.000174	67624
bilirubin_tot_log	mpgs	Pt_5e-08	-0.0178	2.72e-08	-0.0159	7.19e-06	67624
apoA	mpgs	Pt_0.005	-0.0184	1.54e-07	-0.0091	0.873	346
ldl	ipgs	Pt_1e-05	0.0175	1.77e-06	0.0039	0.369	53241
vitD	mpgs	Pt_5e-07	-0.0175	2e-06	-0.0075	0.16	27549
tg_log	vpgs	Pt_1e-05	0.0161	3.23e-06	0.0024	0.522	62937
vitD	vpgs	Pt_0.005	-0.0182	4.94e-06	-0.0075	0.148	27549
ast_log	mpgs	Pt_0.05	0.0161	7.65e-06	0.0125	0.000764	69777
vitD	ipgs	Pt_5e-08	0.0153	4.26e-05	0.0145	0.00549	27549
apoA	vpgs	Pt_5e-05	-0.0144	4.67e-05	0.0065	0.915	346
hba1c	mpgs	Pt_0.001	0.0133	0.000109	-0.0041	0.32	47119
creatinine	ipgs	Pt_0.01	0.0124	0.000125	0.0015	0.621	76321
hscrp_log	mpgs	Pt_0.005	-0.0129	0.000182	0.0125	0.152	11278
cysC_log	ipgs	Pt_0.01	0.0109	0.00088	-0.0052	0.908	573

tg_log	ipgs	Pt_1e-06	0.011	0.00183	0.0126	0.000973	62937
creatinine	mpgs	Pt_5e-08	0.0084	0.00469	0.0021	0.483	76321
ggt_log	ipgs	Pt_5e-08	0.0094	0.00649	0.0059	0.645	5523
ggt_log	mpgs	Pt_0.001	0.0085	0.0106	0.0162	0.214	5523
glu	ipgs	Pt_0.01	0.0101	0.0112	0.0087	0.0119	75604
chol	mpgs	Pt_5e-06	0.008	0.0211	0.0038	0.317	63304
hba1c	ipgs	Pt_5e-08	0.0076	0.0337	0.0059	0.183	47119
ggt_log	vpgs	Pt_1e-06	0.0069	0.0414	0.0066	0.602	5523
glu	mpgs	Pt_0.0005	0.0079	0.0453	0.0093	0.00758	75604
ldl	mpgs	Pt_5e-05	0.0068	0.0473	-0.0028	0.512	53241
glu	vpgs	Pt_1e-05	0.0084	0.0547	-0.0016	0.636	75604
alb	mpgs	Pt_1e-05	0.0066	0.0826	0.002	0.617	60512
hba1c	vpgs	Pt_0.01	0.007	0.162	-0.0074	0.0537	47119
lipA_log	mpgs	Pt_0.0001	-0.0041	0.285	0.0509	0.347	383
alb	ipgs	Pt_0.05	0.004	0.311	0.0016	0.684	60512
creatinine	vpgs	Pt_5e-05	0.0031	0.32	0.001	0.74	76321
cysC_log	mpgs	Pt_5e-07	0.0029	0.362	-0.0164	0.722	573
apoB	vpgs	Pt_0.005	-0.0029	0.442	-0.0321	0.609	437
lipA_log	ipgs	Pt_0.005	-0.0029	0.491	-0.0153	0.828	383
cysC_log	vpgs	Pt_0.005	-0.0022	0.506	0.0495	0.271	573
alb	vpgs	Pt_0.0005	-0.0025	0.509	6e-04	0.876	60512
lipA_log	vpgs	Pt_0.0005	-0.0025	0.543	-0.0076	0.914	383
chol	vpgs	Pt_0.05	0.0022	0.551	-1.00E-04	0.983	63304
ldl	vpgs	Pt_0.0001	-9.00E-04	0.815	-0.0066	0.115	53241

Supp. Table S9: ALT iPGS variant annotation, related to the STAR Methods

SNP	CHR	POS	EA	NEA	beta	P	Gene	Genic location
rs10414043	19	44912456	A	G	-0.00236	5.527e-9	APOE;APOC1	intergenic
rs11735092	4	87305079	C	T	-0.00181	8.576e-11	HSD17B13	UTR3
rs12484530	22	44014113	A	G	0.0038	8.437e-14	PARVB	intronic
rs139052	22	43931132	A	C	0.00256	6.394e-16	PNPLA3	intronic
rs16991199	22	43949005	G	T	0.00523	1.107e-8	PNPLA3;SAMM50	intergenic
rs17036160	3	12288284	T	C	-0.00258	1.007e-9	PPARG	intronic
rs17217098	19	19591575	A	G	0.00328	8.119e-9	PBX4	intronic
rs2642438	1	220796686	G	A	0.00174	5.787e-9	MTARC1	exonic
rs2954021	8	125469835	G	A	-0.00176	1.269e-10	TRIB1;LINC00861	intergenic
rs58542926	19	19268740	T	C	0.0037	8.914e-12	TM6SF2	exonic
rs738408	22	43928850	T	C	0.00655	3.255e-78	PNPLA3	exonic
